# Lectureship on Phrenology

**Published:** 1846-05-01

**Authors:** 


*Lectureship on Phrenology.*—The Andersonian University of Glasgow, Scotland, had by an unanimous vote established a lectureship on phrenology in that institution. Dr. Wm. Weir has been appointed to the chair. Dr. Weir has been a successful lecturer in the medical school of that city, and is one of the physicians to the Glasgow Royal Infirmary.

